# Migratory patterns and connectivity of two North American grassland bird species

**DOI:** 10.1002/ece3.4795

**Published:** 2018-12-26

**Authors:** Jason M. Hill, Rosalind B. Renfrew

**Affiliations:** ^1^ Vermont Center for Ecostudies Norwich Vermont

**Keywords:** *Ammodramus savannarum*, geolocator, migratory divide, partial migration, PinPoint Argos‐GPS tag, population connectivity, *Sturnellamagna*

## Abstract

Effective management and conservation of migratory bird populations require knowledge and incorporation of their movement patterns and space use throughout the annual cycle. To investigate the little‐known migratory patterns of two grassland bird species, we deployed 180 light‐level geolocators on Grasshopper Sparrows (*Ammodramus savannarum*) and 29 Argos‐GPS tags on Eastern Meadowlarks (*Sturnella magna*) at Konza Prairie, Kansas, USA, and six US Department of Defense (DoD) installations distributed across the species' breeding ranges. We analyzed location data from 34 light‐level geolocators and five Argos‐GPS tags attached for 1 year to Grasshopper Sparrows and Eastern Meadowlarks, respectively. Grasshopper Sparrows were present on the breeding grounds from mid‐April through early October, substantially longer than previously estimated, and migrated on average ~2,500 km over ~30 days. Grasshopper Sparrows exhibited strong migratory connectivity only at a continental scale. The North American Great Lakes region likely serves as a migratory divide for Midwest and East Coast Grasshopper Sparrows; Midwest populations (Kansas, Wisconsin, and North Dakota; *n* = 13) largely wintered in Texas or Mexico, whereas East Coast populations (Maryland and Massachusetts, *n* = 20) wintered in the northern Caribbean or Florida. Our data from Eastern Meadowlarks provided evidence for a diversity of stationary and short‐ and long‐distance migration strategies. By providing the most extensive examination of the nonbreeding movement ecology for these two North American grassland bird species to date, we refine information gaps and provide key insight for their management and conservation.

## 1 INTRODUCTION

Effective management and conservation of migratory bird populations require an understanding of their patterns of movement, because individual condition, survival, and breeding performance can be linked by processes that occur across thousands of kilometers and at different stages of the annual cycle (Fretwell, 1972; Norris & Marra, 2007; Renfrew et al., 2013; Rubenstein & Hobson, 2004). Monitoring population‐specific variation in nonbreeding areas, migration routes, and migratory timing can yield important insights into the effects of climate change and local habitat disturbance on population growth, for example, and directly inform conservation measures (Jenni & Kéry, 1988; Martin et al., 2007; Palacín, Alonso, Martín, & Alonso, 2017; Sheehy, Taylor, McCann, & Norris, 2010; Visser, Perdeck, Balen, & Both, 2009). For many species, however, we lack these data, which hinders efforts to implement management actions (Faaborg et al., 2010; Hostetler, Sillett, & Marra, 2011; Sherry & Holmes, 1996; Webster, Marra, Haig, Bensch, & Holmes, 2002).

Migratory connectivity refers to the degree of geographic linkage between populations throughout the annual cycle (Fraser et al., 2012; Marra, Studds, & Webster, 2010; Syroechkovski & Rogacheva, 1995). Populations of long‐distance migrant species frequently have weak migratory connectivity, wherein individuals from a breeding population diffuse across the species' nonbreeding range, mixing with individuals from other breeding populations (Finch, Butler, Franco, & Cresswell, 2017; Fraser et al., 2012). As a result, the influences of localized processes on survival and condition during the nonbreeding period are distributed across many breeding populations (Finch et al., 2017). In contrast, strong connectivity (Cormier, Humple, Gardali, & Seavy, 2013; Hahn, Amrhein, Zehtindijev, & Liechti, 2015) occurs when most individuals from a breeding population overwinter in a geographic area separate from the nonbreeding areas of other breeding populations. In this case, the nature and severity of limiting factors on the migration routes and nonbreeding grounds may vary between populations, perhaps even driving regional population trends on the breeding grounds (e.g., Golden‐winged Warblers [*Vermivora chrysoptera*]; Kramer et al., 2015).

Descriptions of the migratory patterns and nonbreeding areas for North American grassland birds is needed to provide insights into their continental population declines over the past 50 years (Sauer et al., 2017). Despite an intensive conservation focus—largely on the breeding grounds (Askins et al., 2007)—population declines continue; these are attributed to habitat loss and degradation from intensification and expansion of agricultural activities on the breeding and nonbreeding grounds (Askins et al., 2007; Hill, Egan, Stauffer, & Diefenbach, 2018a; Pool, Panjabi, Macias‐Duarte, & Solhjem, 2014). For migratory grassland bird species, basic information about migratory connectivity and key nonbreeding areas could facilitate collaborative conservation efforts and identify factors limiting population growth throughout their annual cycle (Robinson et al., 2010; Webster & Marra, 2005), while miniaturized light‐level geolocators (hereafter geolocators) and GPS tags provide new means of revealing needed data (Bächler et al., 2010; DeLuca et al., 2015). For example, Spoon‐billed Sandpipers (*Eurynorhynchus pygmeus*), a critically endangered species of east Asia, face imminent extinction (Zöckler, Syroechkovskiy, & Atkinson, 2010). Information recently gleaned from tracking tags allowed researchers to discover new stopover and nonbreeding sites, work with local officials to implement restrictions on hunting (a prominent source of mortality in this species), and bolster and expand international conservation efforts from Myanmar to Russia (N. Clark. and R. Green unpublished data; Zöckler et al., 2010). Similarly, analysis of satellite tracking data of Golden Eagles (*Aquila chrysaetos*) revealed that substantial illegal persecution (~one third of tagged eagles) in specific areas of the Highlands was responsible for declining populations in Scotland (Whitfield & Fielding, 2017).

Here, we examine the movement ecology of Grasshopper Sparrows (*Ammodramus savannarum*) and Eastern Meadowlarks (*Sturnella magna*); two species that breed across much of the Midwest and eastern United States have undergone steep declines (≥2.5% per year, 1966–2015; Sauer et al., 2017), but are locally common. Little is known about the migratory behavior of either species, except what has been gleaned from few scattered band recoveries (Jaster, Jensen, & Lanyon, 2003; Vickery, 1996). Grasshopper Sparrows migrate nocturnally, likely within small groups of conspecifics or as individuals, and are difficult to detect outside of the breeding season when on the ground and not singing (Evans & Mellinger, 1999; Vickery, 1996). They winter across the southeastern United States, on some Caribbean Islands, and south to Nicaragua, but migratory connectivity is essentially unknown (Vickery, 1996). Eastern Meadowlark populations may be largely sedentary across much of their range, but available band recoveries (e.g., between Ontario, Canada to South Carolina, and between Indiana and Georgia, US) suggest that some individuals within northern populations are migratory (Jaster et al., 2003). To elucidate the migratory routes and connectivity of these grassland bird species, we deployed geolocators on Grasshopper Sparrows and GPS tags on Eastern Meadowlarks across six US states distributed across their respective breeding ranges.

## 2 METHODS

### 2.1 Study locations

We worked with colleagues at the Department of Defense (DoD) to identify installations harboring populations of grassland birds that would be amenable to our research activities; we chose geographically dispersed installations to allow comparisons across much of the range of Grasshopper Sparrows and Eastern Meadowlarks. In 2015 and 2016, we conducted fieldwork at one nature preserve (Konza Prairie, Kansas; 39.100°N, −96.608°W) and at six DoD installations: Camp Grafton, North Dakota (47.700°N, −98.665°W), Fort Riley, Kansas (39.207°N, −96.824°W), Camp Ripley, Minnesota (46.090°N, −94.358°W), Fort McCoy, Wisconsin (43.967°N, −90.660°W), Joint Base Cape Cod, Massachusetts (41.658°N, −70.521°W), and Patuxent River Naval Air Station, Maryland (38.286°N, −76.408°W). We compared movement patterns of grassland birds between sites in the Midwest (Kansas, North Dakota, Minnesota, and Wisconsin) and the East Coast (Maryland and Massachusetts). All necessary DoD installation, state, and federal permits for wildlife research were obtained prior to fieldwork, and our research protocols followed the Ornithological Council's Guidelines to the Use of Wild Birds in Research (Fair et al., 2010).

### 2.2 Grasshopper sparrow geolocator deployment and recovery

**Figure 1:**
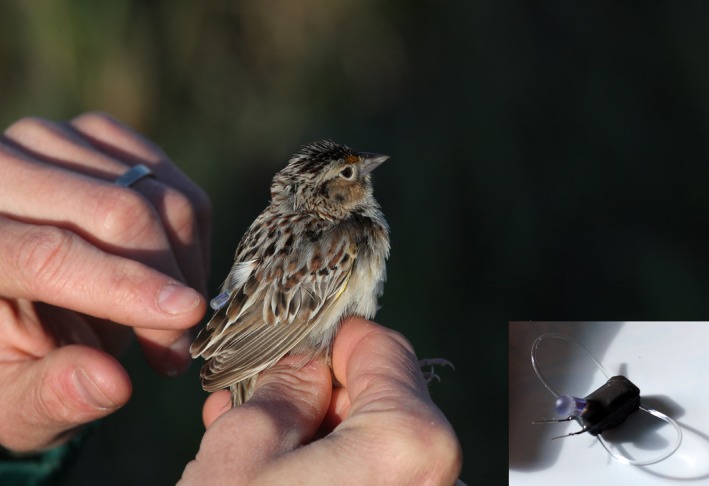
A Grasshopper Sparrow wearing a geolocator (stalk visible at fingertip) prior to release at Camp Grafton, ND, May 2015. The communication wires (for turning on the unit via a laptop software connection) on the geolocator (inset) were trimmed prior to deployment. Photographs by Alex Lehner and Inez Hein (inset)

At each of six DoD installations, we deployed 30 geolocators (model Intigeo‐P50B1‐7‐dip, Migrate Technology Ltd., Cambridge, UK) between 11 May and 18 June, 2015 (median = 26 May), for a total of 180 geolocators deployed. Using mist nets and audio playback, we captured adult male Grasshopper Sparrows on their territories. Sparrows fitted with a geolocator received a unique combination of leg bands: three colored plastic and one USGS aluminum. Geolocator harnesses were constructed from an 81‐mm section of Stretch Magic jewelry cord (0.7 mm) passed through two transverse tubes embedded in the plastic geolocator housing. We then melted the jewelry cord ends together to form a single circular loop divided by the geolocator (Figure 1, inset). A geolocator (~0.52 g) and harness together weighed ≤3.0% of each sparrow's body mass and were positioned on the lower back via a leg loop harness (Figure 1). We verified a good harness fit by ensuring that ~2 mm of vertical play occurred when we gently lifted the geolocator from a bird's back, or else we replaced the harness. Geolocators sampled light intensity at 1‐min intervals and recorded the maximum light intensity every 5 min.

In 2016, we systematically searched for color‐banded Grasshopper Sparrows wearing geolocators over a similar time period as our visits in 2015. We concentrated our searches on the territorial locations of males captured in 2015 and then expanded our search area outward as time permitted. At two installations (Forts Riley and McCoy), military training exercises or the risk of unexploded ordinance prevented us from searching the entire 2015 study area. We recaptured male sparrows with mist nets deployed within flight lanes and used audio playback when netting alone was unsuccessful. We examined all recaptured males for signs of injury or discomfort that could be associated with the geolocator or harness. We used a paired Bayesian *t* test to compare bird capture weights in 2015 to recapture weights (without geolocator and harness) in 2016 with the BayesianFirstAid package in R; this test assumes a bivariate *t* distribution, as opposed to a bivariate normal distribution, and is less sensitive to outliers compared to a frequentist *t* test (Bååth, 2014; R Core Team, 2018).

### 2.3 Eastern meadowlark GPS tag deployment, data recovery and analysis

We captured 29 male Eastern Meadowlarks on their breeding territories in 2016 using mist nets between 21 April and 11 June at four DoD installations (Fort Riley [*n* = 5], Fort McCoy [*n* = 10], Joint Base Cape Cod [*n* = 7], and Patuxent River Naval Air Station [*n* = 4]) and Konza Prairie [*n* = 3]. We constructed leg loop harnesses out of a continuous piece of Stretch Magic jewelry cord (1.0 mm) to affix a 4.0‐g PinPoint Argos‐GPS tag (Lotek Wireless, Canada), with an 18‐cm antenna (hereafter GPS tag) reinforced at the base to guard against bird‐inflicted damage. Harnesses were constructed on the bird and finished with a single double‐overhand knot next to the GPS tag; the knot was also covered in a thin film of Loctite super glue (Henkel Corporation, Connecticut, USA). We assessed harness fit as we did for Grasshopper Sparrows. A GPS tag and harness together weighed ≤4.0% of each meadowlark's body mass.

GPS tags had a memory capacity to store 30 GPS fixes (i.e., location estimates) with an accuracy of ~10‐m (M. Vandentillaart pers. comm.). We programmed GPS tags to collect weekly fixes from September to November and February to April and once per 2 weeks for the rest of the period between 15 July 2016 and 15 April 2017 (see Hill & Renfrew, 2018b for complete list of dates); GPS fixes were attempted at 12:00 UTC. GPS data were stored onboard the tags until a preprogrammed date (15 April 2017) when all data were to be uploaded concurrently to the Argos satellite system. A GPS tag would not have transmitted any data, therefore, if the tag had malfunctioned or became damaged prior to 15 April 2017.

### 2.4 Geolocator light‐level data processing

All data from geolocators were downloaded by Migrate Technology Ltd, and we discarded light‐level data once the units started consistently recording maximum light values, which indicate that the battery is near the end of life (J. W. Fox pers. comm.). We used package BAStag (with a light threshold value of 1.0) within program R to estimate daily twilight times from geolocator light‐level data (Hill & Braun, 2015; Wotherspoon, Sumner, & Lisovski, 2016). It has been common practice to edit twilight times based on a visual inspection of the geolocator data, but this practice potentially reduces scientific repeatability (Bridge et al., 2013). Therefore, following Cooper, Hallworth, and Marra (2017), we only edited twilight times in egregious examples, such as when BAStag estimated that a twilight event occurred during the middle of the day or night (*n* = 9 out of 18,952 twilight events [0.05%]). BAStag also rarely (<10 times) estimated two twilight events within minutes of each other; in those instances, we visually selected a twilight event closest to the threshold value. Unedited geolocator data are available at Movebank (Hill & Renfrew, 2001; Wikelski & Kays, 2018).

We converted the timing of estimated twilight events into estimates of latitude and longitude using package FLightR (Rakhimberdiev & Saveliev, 2016). FLightR uses a particle filter algorithm within a Bayesian framework to combine a random walk movement model (with two states: *sedentary* or *migrating*), a hidden Markov model that probabilistically estimates unobserved animal locations, and an optional user‐defined spatial probability mask based on a 50 × 50‐km grid (Rakhimberdiev et al., 2015). Each grid cell is categorized as *land* or *water*, and the spatial probability mask allows the user to define movement rules separately for these two cell types. We ran FLightR with 1 million particles, the outlier routine turned on, and a twilight movement prior of 0.05 because Grasshopper Sparrows are sedentary for most of the year (Vickery, 1996). We chose a migration direction prior of 180° (i.e., due south) for dates May–December 2015 and 0° (i.e., due north) for dates January–May, 2016 (Vickery, 1996). We constrained the movement model to allow up to 810‐km flights based on a maximum 15‐hr flight between twilights at an assumed maximum flight speed of 54.0 km/hr (Pennycuick, Åkesson, & Hedenström, 2013).

Our spatial probability mask allowed location estimates up to 1,500 km from shore, because Grasshopper Sparrows are regular vagrants to Bermuda (Vickery, 1996), and from 49.0°N to the equator and between −110.0°W and −60.0°W. To accommodate for the coarseness of the FLightR spatial grid, we treated nearshore areas (≤25.0 km from the coastline) as land and allowed Grasshopper Sparrows to remain stationary over offshore waters (>25.0 km from the coastline) with only a 5% probability (Cooper et al., 2017). In preliminary analyses, the FLightR model results suggested that some birds moved frequently (often back‐and‐forth) between Caribbean Islands and Florida during winter; similar patterns of unlikely movement behavior had been previously reported for Kirtland Warbler (*Setophaga kirtlandii*) in the Caribbean based on geolocator data (Cooper et al., 2017). To avoid similar model behavior, we followed the approach of Cooper et al. (2017) by treating 300 km around the Cayman Islands, Cuba, and Jamaica as nearshore areas (i.e., land). For offshore location estimates, such an approach does not coerce location estimates onto land. The resulting estimates of migration routes, flight speed, and timing of migration were indistinguishable from our preliminary analyses.

Individual geolocators record light with different levels of precision, and the environmental conditions experienced by each sparrow affect the amount of light that reaches the geolocator (Fudickar, Wikelski, & Partecke, 2012; Lisovski et al., 2016). Therefore, FLightR uses a user‐defined calibration period to model the relationship between the observed and expected light levels for a given location (Rakhimberdiev et al., 2015). For each bird, we used a calibration period spanning from the day after it was banded through 1 August 2015 (median = 57 days, 44–80 days); during this time period, we assumed the bird was present on its breeding territory. Geolocators attached to three sparrows remained functional until shortly after they returned to their breeding grounds in 2016 (as estimated by our FLightR model), which allowed us to use a second calibration period. For these three sparrows, we reran the FLightR model with a second calibration period (medium = 6 days, range = 5–10 days) representing the time period when the birds were back on their breeding grounds in 2016 with functioning geolocators. Including a second calibration period for these three birds allowed us to use the FLightR tag aging model, which linearly accounts for the increased opaqueness (and decreased sensitivity) of the transparent shell of the light sensor that occurs over long periods of time.

### 2.5 Geolocator statistical analysis

To identify stationary (≥2 consecutive twilights) and movement periods throughout the year for each bird, we used the stationary.migration.summary function in FLightR. Following Hahn et al. (2015) and Jacobsen et al. (2013), we combined stopover events during migration that were <45 km apart due to the spatial resolution of the data (see Results). Accordingly, we present stopover frequency and duration as integers. We identified the onset of fall migration as the first movement of at least 45 km (the default FLightR minimum distance for movement) south of the breeding grounds (Hewson, Thorup, Pearce‐Higgins, & Atkinson, 2012). We considered arrival on the nonbreeding grounds to have occurred when a bird stopped moving in a southerly direction consistent with fall migration (Fraser et al., 2012). We identified the start of spring migration by identifying the movement period that carried the bird >45 km northward from its nonbreeding grounds, after which the bird continued to move northward.

We measured the length of migration routes by connecting consecutive median twilight location estimates between FLightR‐identified stationary periods with a great‐circle path between the breeding and first stationary period on the nonbreeding grounds (fall migration) or ultimate nonbreeding stationary period location and breeding grounds (spring migration). For each sparrow, we calculated migration speed (km/day; migration distance traveled divided by total days) and traveling rate (km/day; migration distance traveled divided by number of days when the bird was nonstationary). For sparrows whose geolocator functioned until the onset of spring migration, we created 50% and 90% kernel utilization distributions (UD) for the complete nonbreeding periods in FLightR and calculated their area in ArcGIS (ESRI, 2016). Our approach explicitly incorporates nonbreeding location uncertainty in the estimation of the UD; see (Hill & Renfrew, 2001) for FLightR‐related R code complete with tutorial.

### 2.6 Grasshopper sparrow migration connectivity

We assessed the strength of migration connectivity (MC) with the MigConnectivity package in R which uses a matrix populated with distances between nonbreeding (Texas, Mexico, or Florida‐Caribbean in our study) and breeding areas (Cohen et al., 2017). We also report the distances (km) between individuals from the same population during the nonbreeding period (Finch et al., 2017), an MC estimate combining all populations, and the MC estimate for only the Midwest populations. We could not produce an MC estimate for only the East Coast populations, because all East Coast birds overwintered within Florida or the Caribbean; winter location errors for most birds overlapped both regions (see Results).

### 2.7 Location accuracy and general statistical analysis

As a measure of location accuracy, we measured the distance between each bird's known territory and the FLightR model's location estimates throughout the calibration period in 2015. Likewise, we calculated the half‐width of the 95% credible interval (CRI) for locations during January as a measure of nonbreeding location precision; interval half‐widths are commonly used to measure uncertainty of intervals (e.g., Phillips & Gregg, 2001). To visually demonstrate location uncertainty, we created a composite error polygon for each sparrow during fall and spring migration. For each estimated location (i.e., at each twilight) during migration, we created a minimum convex polygon (MCP) using the upper and lower limits of the 95% CRI for latitude and longitude. We then created a composite error polygon by overlaying all MCPs for each sparrow during fall or spring migration. Statistical results are presented as (median [$\tilde{x}$] ± *SD*, range [min–max]) unless otherwise noted.

## 3 RESULTS

### 3.1 Grasshopper sparrow fall migration

**Figure 2:**
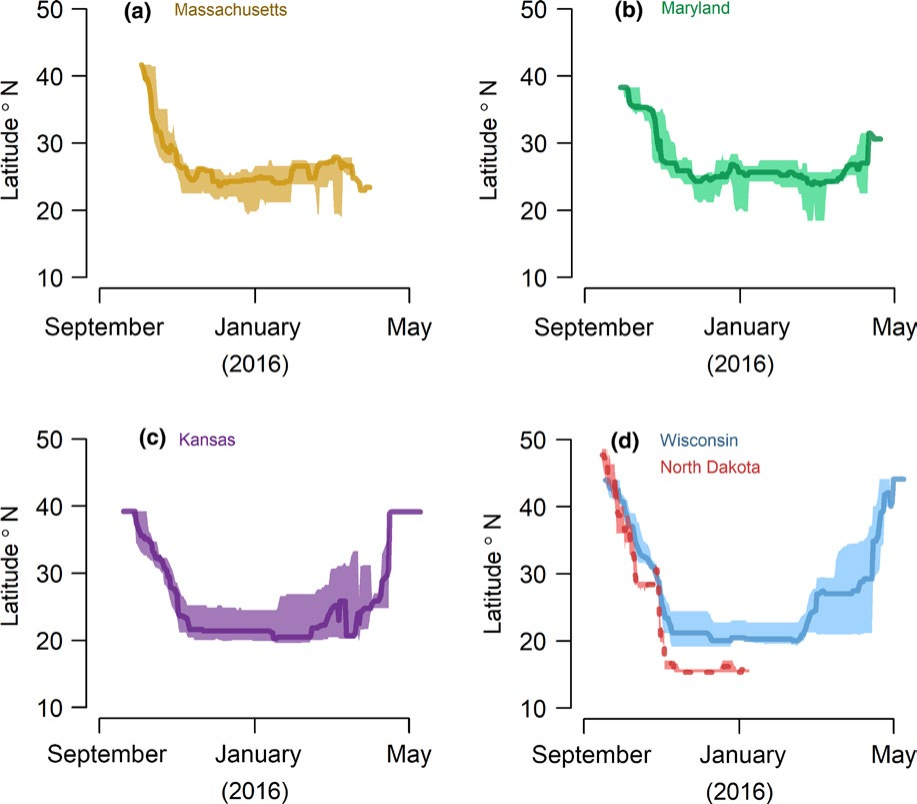
Median latitude (dark line) and interquartile range (shaded area) from the onset of fall migration onwards for all Grasshopper Sparrows that were initially fitted with a geolocator in Massachusetts (*n* = 10, panel a), Maryland (*n = *10, panel b), Kansas (*n = *8, panel c), Wisconsin, (*n* = 4), and North Dakota (*n* = 1, panel d), 2015–2016. Sample sizes refer to maximum sample size for that population, as sample size changed on a daily basis

**Figure 3:**
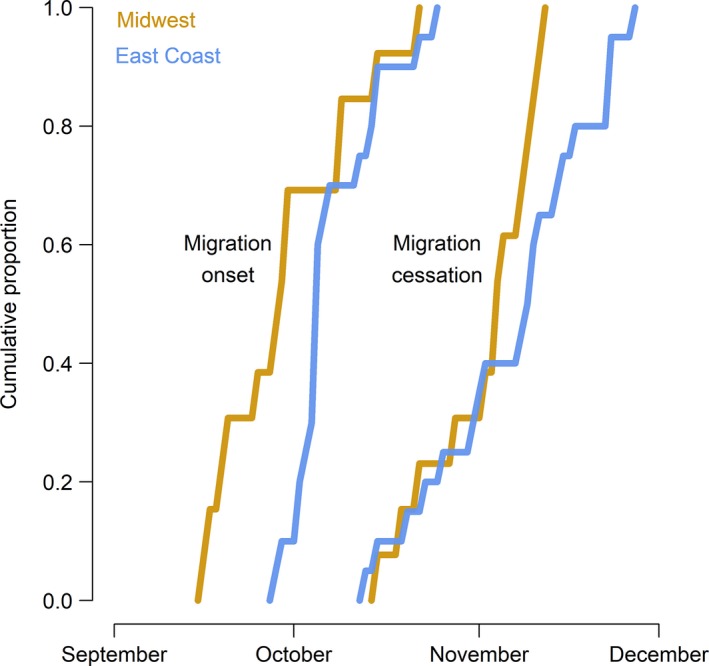
Cumulative proportion of Grasshopper Sparrows that started fall migration (migration onset) and reached the nonbreeding grounds (migration cessation), by date and breeding population: Midwest (brown: Kansas, North Dakota, and Wisconsin, *n* = 13 sparrows) and East Coast (blue: Maryland and Massachusetts, *n* = 20 sparrows)

**Figure 4:**
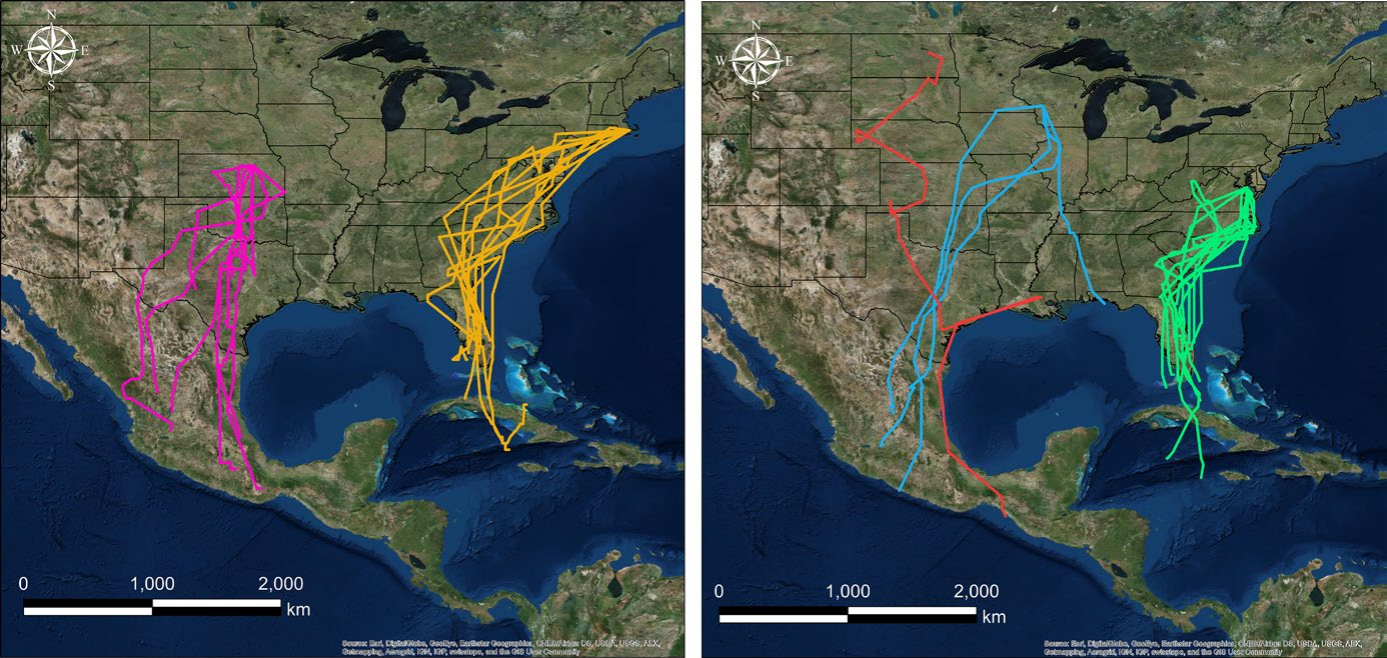
Probable fall migration routes for Grasshopper Sparrows from breeding populations in Kansas (purple, *n* = 8) and Maryland (green, *n* = 10; left panel) and North Dakota (red, *n* = 1), Wisconsin (blue, *n* = 4), and Massachusetts (orange, *n* = 10; right panel). Routes were created by connecting consecutive twilight location estimates with orthodromic lines

In 2016, we retrieved 35 geolocators (19.4%) from recaptured Grasshopper Sparrows that provided usable data for a median of 287 days (±53.1 days, 19–338 days, *n* = 34; see Supporting Information Table S1); data from one geolocator were inadvertently lost prior to data analysis. Sparrows typically delayed fall migration until after September (5 October ±9.6 days, 16 September–25 October 2015, *n* = 33). Over the course of a month, typically (30.0 days± 15.2, 8.5–58.5 days; Figures 2 and 3), sparrows traveled ~2,500 km (2491.73 km ±895.80, 1147.71–6291.29 km) to their nonbreeding grounds. Stopovers were infrequent (4.0 ± 2.00, 1.00–9.00) and typically short (3.0 days ± 5.0, 2.0–32.0 days). Median fall migration speed was 82.27 km/day (±62.29, 32.42–314.11 km/day), and median travel rate was 153.27 km/day (±152.14, 33.21–748.16 km/day). Fall migration routes were almost entirely over land for all but five Grasshopper Sparrows (from the Massachusetts and Maryland study populations) that likely wintered in the Caribbean via passage through southern Florida (Figure 4). Sparrows from Midwest populations commenced fall migration ~2 weeks before sparrows from the East Coast (Figure 3).

### 3.2 Grasshopper sparrow connectivity

The Grasshopper Sparrows in our study wintered at a wide range of elevations and habitats from Caribbean Islands, to the Chiapas coast of Mexico, to the Southern Sierra Madre and Central Plateau of Mexico (Hill & Renfrew, 2001). Grasshopper Sparrows from Midwest populations wintered in Mexico (*n* = 10), Texas (*n* = 2), or the Florida panhandle (*n* = 1), whereas East Coast sparrows (*n* = 20) wintered in Florida, the Greater Antilles, or possibly the Bahamas (Figures 4 and the course of a month, typic5; See Supporting Information Figure S1). The 95% CRI half‐widths of locations during January equated to ~165 km (latitude) and ~50 km (longitude). When all breeding populations were included, results from the migratory connectivity test suggested strong migratory connectivity at the continental scale between breeding and nonbreeding areas (MC = 0.81, *n* = 33). However, individuals from the same breeding population wintered a median of 473.05 km apart (± 425.97, 39.74–1942.71 km), and Midwest birds were twice as far apart from each other as East Coast birds (see Supporting Information Table S2). No regional migratory connectivity was detected when only sparrows from the Midwest were considered (MC = −0.02, *n* = 13).

**Figure 5:**
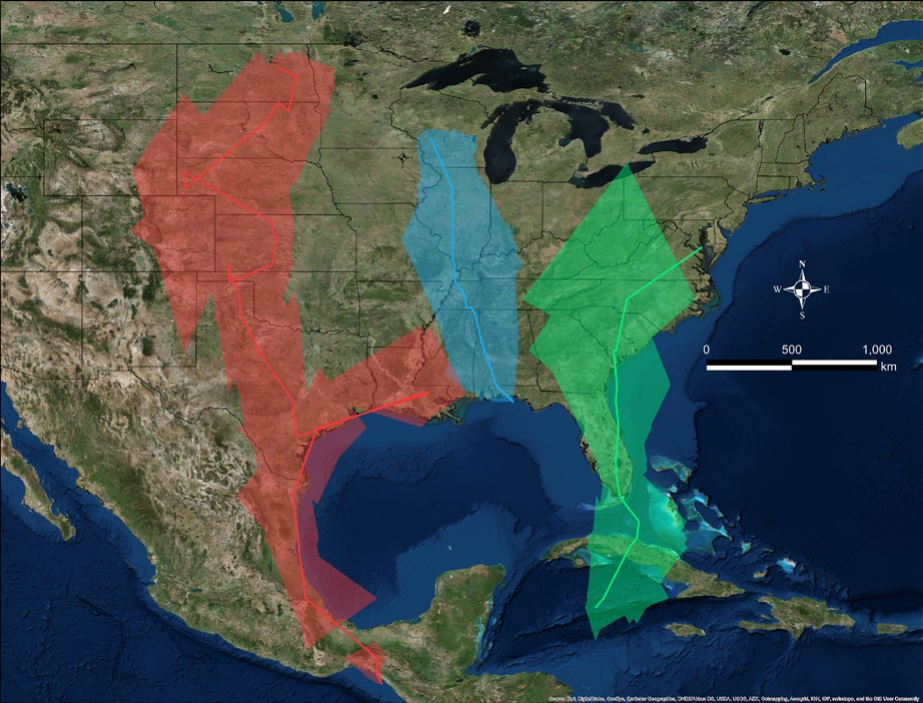
Examples of probable fall migration routes (dark lines) and the uncertainty of location estimates (shaded regions) for three Grasshopper Sparrows fitted with geolocators in North Dakota (red), Wisconsin (blue), and Maryland (green), respectively. Routes were created by connecting consecutive twilight median location estimates with orthodromic lines. Error polygons (shaded regions) were created by overlaying twilight‐specific 95% CRIs during fall migration (see Methods)

### 3.3 Grasshopper sparrow spring migration

Most geolocators ceased to function over the winter months, but 12 sparrows with functioning geolocators started spring migration (8 March ±22.16 days, 15 January–30 March 2016); three geolocators were still functioning when birds arrived at the breeding grounds on 17 April, 20 April and 2 May 2016, respectively (Figure S2). All three of these sparrows (i.e., with a completely recorded spring route) followed a counter‐clockwise elliptical loop migration pattern: Spring paths were farther east than fall migration paths (Hill & Renfrew, 2001). Cumulative annual migration distance (fall + spring) for the three birds was a median of 5196.93 km (±1644.88, 3325.11–6603.90 km).

### 3.4 Grasshopper sparrow annual cycle

**Figure 6:**
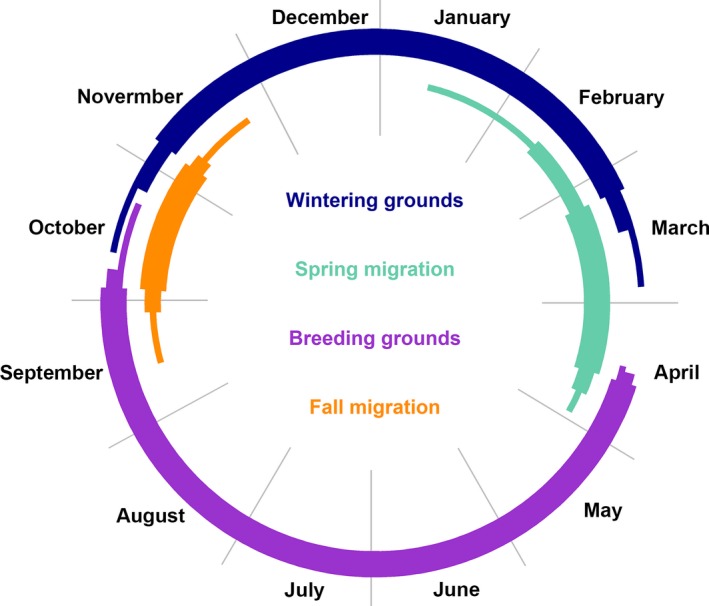
Annual cycle of Grasshopper Sparrows (*n* = 33), as estimated from geolocator data. Each color represents a period of the annual cycle, and the data for each period are presented by three arcs (from thickest to thinnest) drawn from the median to median, 25th to 75th percentile, and minimum to maximum date, respectively. Sample size varies throughout the annual cycle

Fall migration spanned 30.00 days (±15.22, 8.50–58.51 days) of the total annual cycle for the 33 sparrows that completed fall migration; the peak of migration occurred between 9 and 28 October, when >75% of these sparrows were engaged in migration (Figure 3). The 12 sparrows (that carried functioning geolocators until the start of spring migration) spent ~3–5 months (127.49 days ± 23.25, 83.99–153.99 days) of the annual cycle on their nonbreeding grounds. The 50% kernel UDs for the entire nonbreeding period covered a median of 9231.97 km^2^(±16581.10, 6106.40–57572.04 km^2^, *n* = 12), including the uncertainty of location estimates. Sparrows were largely stationary at the nonbreeding grounds, spending only about a week (7.08 days ± 24.53, 0.00–87.67 days, *n* = 12) on the move. Spring migration duration varied substantially between the three sparrows that completed spring migration with functioning geolocators; they spent a median of 35.00 days (±28.43, 17.99–73.51 days) and 161.00 days (±8.32, 148.01–163.48 days) of their annual cycle on spring migration and the breeding grounds, respectively (Figure 6).

### 3.5 Grasshopper sparrow condition and return rates

We recaptured all but one color‐banded sparrow that we relocated in 2016, and all recaptured birds were still wearing their geolocators. Return rates varied widely (overall = 0.19, range = 0.00–0.40) across all six DoD installations (see Supporting Information Table S1). We detected no serious injuries on recaptured birds, but five birds developed ~1–2 mm of skin overgrowth covering parts of the harness in the inner thighs (similar to reports by Arlt, Low, & Pärt, 2013); we were able to easily extract the embedded harness material. All five of these birds maintained territories in 2016 and appeared to be unaffected by the skin overgrowth. Most birds lost feathers directly beneath the geolocator, and one bird had an enlarged preen gland (possibly due to rubbing from the geolocator). At the time of recapture, most geolocator harnesses fit loosely with ~1 cm of vertical play when the geolocator was lifted vertically from the birds' backs. Body mass of Grasshopper Sparrows in 2016 was not different from initial capture weights in 2015 (mean difference [2016–2015 weight] = +0.24 g, *SD* = 0.71 g, 95% CRI: −0.11 to 0.50 g, *n* = 35). FLightR location estimates of sparrows through the end of the calibration period were a median of 21.80 km (*SD* = 35.64 km) from the sparrows' known territory locations, suggesting high precision of locations during the breeding season.

### 3.6 Eastern meadowlark partial migration, movements, and nonbreeding areas

**Figure 7:**
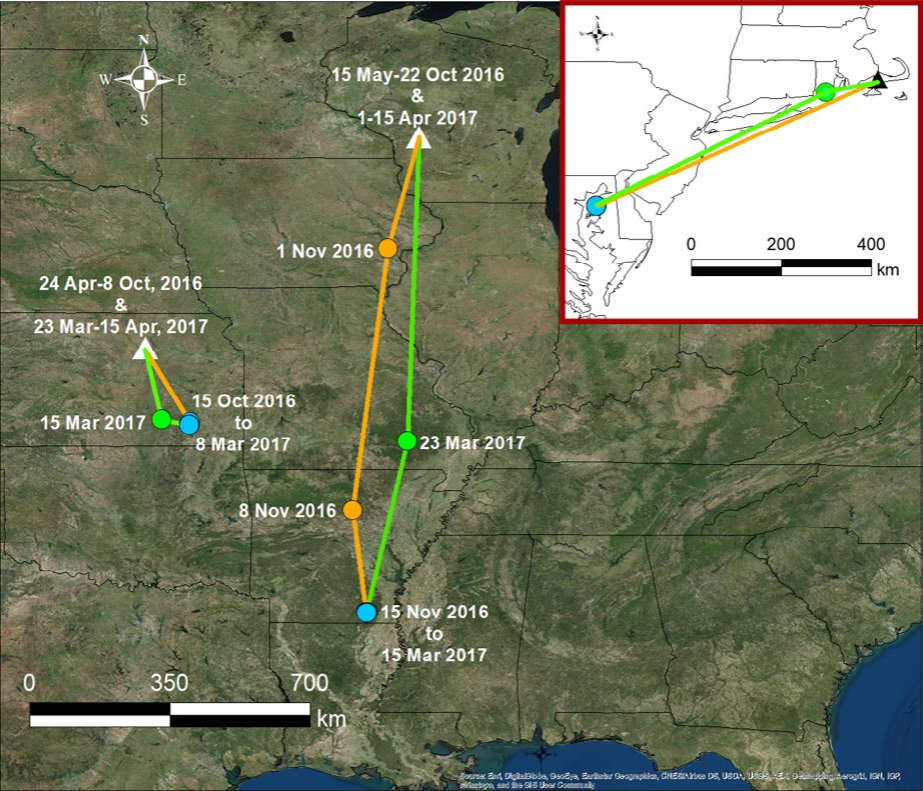
GPS tag locations (circles) during fall (orange) and spring (green) migration, and the nonbreeding period (blue) from Eastern Meadowlarks originally tagged at Department of Defense installations (triangles) in Kansas and Wisconsin (main panel), and Massachusetts (inset). Colored lines are orthodromic lines between consecutive (~7–14 days apart) locations. Location estimates were obtained for the meadowlark tagged in Massachusetts on the breeding (29 April–22 October 2016 and 15 March–15 April 2017) and nonbreeding (15 November 2016–1 March 2017) grounds, and during spring migration (8 March 2017). Not shown are two meadowlarks tagged in Maryland that were non‐migratory year‐round residents

We obtained location data from 11 of 29 deployed GPS tags on Eastern Meadowlarks (Hill & Renfrew, 2018b), and the migration patterns varied substantially between individuals. Five GPS tags provided location estimates of meadowlarks throughout the reporting period (Figure 7): (a) two meadowlarks from Patuxent River Naval Air Station were year‐round residents of the airfield; (b) one meadowlark from Joint Base Cape Cod migrated ~556 km in early November and early March to and from Maryland; (c) one meadowlark from Fort Riley migrated ~328 km in mid‐October and mid‐March to and from southeast Kansas via a clockwise loop; and (d) one meadowlark from Fort McCoy migrated 1,201 km to southeast Arkansas in late October and mid‐March via a counter‐clockwise loop. As assessed via aerial photography, Eastern Meadowlarks predominantly used agricultural grasslands (e.g., hayfields) throughout their migration and nonbreeding periods (Hill & Renfrew, 2018b).

Of the remaining six tags, one malfunctioned soon after deployment and provided no useable data. Three tags reported data from a likely stationary location (±10 m), suggesting that they were on the ground throughout the entire July–April reporting period. Another tag provided only five locations, and another tag provided three location estimates followed by 21 stationary location estimates. For tags that transmitted from a stationary location, crews searched ground cover on multiple occasions for carcasses or tags at the stationary location during the summer of 2017, but were unable to locate the tags. We also learned of the fate for another meadowlark whose tag did not transmit data. A lone hunter witnessed and reported a group of hunters who shot and killed a GPS‐tagged meadowlark on 23 October 2016, at Crane Wildlife Area, Massachusetts (adjacent to Joint Base Cape Cod). The lone hunter recovered the carcass, but was unable to locate the GPS tag which was likely removed by the group of hunters.

## 4 DISCUSSION

### 4.1 Annual cycle and migratory connectivity

Our results provide the most comprehensive assessment to date of the annual cycle and migratory behavior of Grasshopper Sparrows and Eastern Meadowlarks. The Grasshopper Sparrows in our study spent nearly half the year on their breeding grounds; they utilized a strategy of delayed fall migration by remaining on or near breeding sites until early October, even though fledging from nests typically occurs by early August (Hill & Diefenbach, 2014; Hovick et al., 2017; Sutter & Ritchison, 2005). Sparrows commenced spring migration by 1 April and returned to breeding sites from 17 April through 2 May. Our findings differ substantially from the birds of North America account for this species, which used anecdotal reports to estimate that northern populations predominantly migrated south in August and September and commenced spring migration in May (figure 4 in Vickery, 1996). We suspect that the secretive nature of ground‐dwelling male Grasshopper Sparrows, when not singing, allows them to pass undetected before and after their peak breeding period, and (color‐banded) female sparrows are rarely detected during breeding season surveys (Hill, 2013).

Although the reasons are unclear, bird species of open habitat (e.g., Grasshopper Sparrow, Savannah Sparrow [*Passerculus sandwichensis*], and Scissor‐tailed Flycatcher [*Tyrannus forficatus]*) typically delay fall migration longer (~October) and spend a greater proportion of the year (>40%) on their breeding grounds than species of other habitats in North America (this study; Jahn et al., 2012; reviewed in McKinnon, Fraser, & Stutchbury, 2013; Woodworth et al., 2016). Climate change, specifically warming local temperatures in autumn, has been suggested as a long‐term driver of this pattern for many short‐distance migrant species (Jenni & Kéry, 1988; Lehikoinen & Jaatinen, 2012). It is unclear, however, why grassland bird species would be more affected than birds occupying other habitats, especially considering that northern grasslands appear to be rather resilient (in terms of net productivity, alpha diversity, and community composition) to climate change (Craine et al., 2013; Grime et al., 2013).

High relative isolation during any part of the annual cycle potentially subjects a greater proportion of a species' population to processes that limit survivorship (Cooper et al., 2017; McFarland et al., 2013). Grasshopper Sparrows in our study exhibited weak migratory connectivity within regions, and strong migratory connectivity only from a continental scale. Our results, and those of others, point to the need to evaluate migratory connectivity from a hierarchical perspective, especially in the context of conservation and management (Cooper et al., 2018; Fraser et al., 2012; Trierweiler et al., 2014). For example, Martin et al. (2007) used an optimal search algorithm to allocate funds to conserve American Redstarts (*Setophaga ruticilla*) across their breeding range by purchasing lands on the breeding and/or nonbreeding grounds. When they removed information about migratory connectivity from the model, the optimal strategy to bolster the species overall unintentionally resulted in the near‐extinction of some regional populations. Bird populations may also be subjected to limiting factors through mortality associated with particular migration routes stemming from environmental (e.g., drought) and anthropogenic conditions (Hewson et al., 2012; Trierweiler et al., 2014; Whitfield & Fielding, 2017).

Migratory bird species with relatively restricted winter ranges are more likely to experience declines than species with broad nonbreeding ranges, which suggests that species with strong migratory connectivity are more susceptible to the effects of local nonbreeding habitat loss and degradation (Fuller, 2016; Gilroy, Gill, Butchart, Jones, & Franco, 2014; Sutherland, 1996). However, habitat loss and disturbance at regional scales may influence population trends. For example, Golden‐winged Warbler (*V. chrysoptera)*populations exhibit strong migratory connectivity across the species' breeding range. Populations in the upper Midwest of the United States are relatively stable and largely winter in Central America, while the Appalachian population is declining and mainly winters in northern South America (Kramer et al., 2015; Rosenberg et al., 2016). The difference in these trends may be due to the relatively greater recent losses of forest‐dominated landscapes in northern South America (Kramer et al., 2015). The differences in regional population trends of many other North American passerines may also be explained by migratory connectivity patterns (Kramer et al., 2015), but Grasshopper Sparrow migratory connectivity patterns do not closely align with their observed breeding population trends (Sauer et al., 2017). Differences in the annual range of these two sparrow populations correspond approximately with the ranges of two migratory Grasshopper Sparrow subspecies *Ammodramus savannarum pratensis* (East Coast population) and *A. s. perpallidus* (Midwest population), although the two subspecies' breeding ranges overlap in the Midwest (Vickery, 1996).

### 4.2 Migratory divides and fall migration strategies

Migratory divides are common among migrant songbird populations (Finch et al., 2017), and the Great Lakes region likely serves as a divide (Salomonsen, 1955) between central and East Coast populations of Grasshopper Sparrows (this study), American Robins (*Turdus migratorius*; Brown & Miller, 2016), American Redstarts (Norris et al., 2006), and possibly many other species (Kramer et al., 2015). In particular, the migratory patterns of American Robins, elucidated through band recoveries, closely resemble those of Grasshopper Sparrows in our study. American Robins west of the (approximately) Mississippi River predominantly migrated to Texas, while robins (approximately) east of the Mississippi River mostly migrated to the southeastern United States. Furthermore, robins that overwintered in Florida originated largely from coastal breeding populations, whereas robins breeding in Wisconsin wintered in Texas and the southeastern United States (Brown & Miller, 2016); these patterns are similar to our results.

Even within a population, the timing of fall migration varies between years and is likely related to favorable weather (surface air pressure and wind direction) and local declines in temperature and ecological productivity (Ellwood, Gallinat, Primack, & Lloyd‐Evans, 2015; La Sorte et al., 1943; Schmaljohann, Lisovski, & Bairlein, 2017). In our study, Midwest sparrows commenced migration earlier than East Coast sparrows (Figure 3), but we do not know whether this difference in timing occurs consistently across years. Macdonald et al. (2012) used geolocators to monitor the migration of 13 Snow Buntings (*Plectrophenax nivalis*) from Nunavut, Canada, in two consecutive years. Buntings departed from the breeding grounds between 23 September and 6 October in the first year, and between 8 and 10 October in the second year. Mean fall migration departure date for populations of Barn Swallows (*Hirundo rustica*) in Washington State, United States, and Saskatchewan, Canada, varied by nearly a month between years (Hobson et al., 2015). Similarly, Wood Thrush (*Hylocichla mustelina*) fall migration timing (e.g., departure date and duration) is highly variable across years (Stanley, MacPherson, Fraser, McKinnon, & Stutchbury, 2012).

Although we obtained fewer data than we hoped for Eastern Meadowlarks, these depicted the species' partial migration behavior: strategies varied from year‐round residency to short‐ and long‐distance migration. Partial migration is a widespread characteristic of animal migration, in which populations consist of resident and migratory individuals (Lack, 2012); it has been documented in species such as Blue Tits (*Cyanistes caeruleus*), Northern Flickers (*Colaptes auratus*), and American Robins (Brown & Miller, 2016; Gow & Wiebe, 2008; Smith & Nilsson, 1987). Multiple factors including competition for resources or mates and predation risk have been linked to partial migration, but the decision to stay or migrate is largely dependent on individual condition (Chapman, Brönmark, Nilsson, & Hansson, 2011). Due to the poor performance of the GPS tags on meadowlarks, however, we obtained data from too few individuals to assess migratory connectivity or potential causes of migration decisions. Perhaps Eastern Meadowlarks are poor candidates for wearing tracking devices; in our study, two meadowlarks were observed aggressively picking at the antenna immediately following deployment. After we had purchased our GPS tags, Scarpignato et al. (2016) published their difficulties with 3.4 g PinPoint GPS tags that they deployed on three shorebird species; only four of 38 tags (10.5%) communicated data. Improved solar‐powered real‐time GPS tag technology or stable isotopes collected from feathers on the nonbreeding grounds may provide a more complete assessment of migratory connectivity.

### 4.3 Implications for conservation and management

Elucidating the temporal and spatial connection between migratory bird populations, and identifying limiting factors associated with regional population trends, is needed to develop regional conservation strategies (Macdonald et al., 2012; Marra et al., 2010; Martin et al., 2007). Given the strong continental migratory connectivity of Grasshopper Sparrows and the partial migration behavior of Eastern Meadowlarks, our research highlights the need for regional management strategies that consider the full annual cycle of these two grassland‐dependent bird species. For Grasshopper Sparrow, its relatively short daily fall migration flights and its ability to rapidly discover newly‐created small pockets of habitat (Andrews, Brawn, & Ward, 2015; Hill & Diefenbach, 2014), suggest that the species may benefit from many scattered parcels of habitat throughout its migration corridor, as opposed to a few isolated reserves of large grasslands.

Our data suggest that lands supporting breeding populations of Grasshopper Sparrows can continue to host these populations until southbound migration commences, by avoiding intensive activities such as burning or disking until mid‐October. Breeding ground management for grassland birds has traditionally been heavily focused on the nesting season (Askins et al., 2007), with the understanding that migration commences shortly after the nesting season ends in early August (Hill & Diefenbach, 2014). Our study, however, identifies an opportunity to benefit Grasshopper Sparrows beyond the nesting period for several months by, for example, identifying and manipulating vegetation conditions associated with abundant food resources and high survival. For both species, an extended inter‐state or international collaborative approach is likely needed to identify and manipulate habitat conditions that improve individual condition and increase survival during the nonbreeding season.

Our research more narrowly defines information gaps outside of the breeding season for these two species, but we also identified several challenges to further revealing their nonbreeding habitat use and movements. Like most geolocator studies, our results would benefit from confirmation of movement behavior, especially during August and September, via direct observation. Low site fidelity slows the process of information discovery from geolocator studies. Grasshopper Sparrow return rates tended to decline from the East Coast to the northern Midwest, consistent with other studies on this species (Kaspari & O'Leary, 2018; Small, Parks, Gruber, & Gill, 2009). Obtaining year‐round location data via geolocators for sparrow populations in the Upper Midwest will require substantially greater search efforts, although deployment of geolocators (Hallworth, Sillett, Wilgenburg, Hobson, & Marra, 2016) or a feather stable isotope approach on the nonbreeding grounds may prove more efficient. Describing the stopover ecology and nonbreeding habitat use by both species, especially for Grasshopper Sparrows in the Caribbean Islands, should be a priority for future research. Given the variability of migration strategies among individual meadowlarks, tagging territorial males at random seems inefficient. Future studies should explore links between Eastern Meadowlark migration strategies and individual condition, social status, and reproductive performance (Chapman et al., 2011), in order to predict which individuals will migrate and to target those individuals for tagging and tracking.

## AUTHORS' CONTRIBUTIONS

RBR conceived of the project and secured funding, and JMH coordinated the overall research project. JMH and RBR contributed to data collection and writing of the manuscript. JMH processed and archived data, conducted the statistical analyses, and created figures.

## DATA AVAILABILITY

Grasshopper Sparrow light‐level geolocator data (raw and processed) and R script, (Hill & Renfrew, 2001) and Eastern Meadowlark GPS tag locations (Hill & Renfrew, 2018b) are deposited at Movebank.
