# Supplementary figures and images for: Migratory patterns and connectivity of two North American grassland bird species
Source: Ecol Evol. 2018 Dec 26;9(1):680–92. doi: 10.1002/ece3.4795 (PMC6342103; doi:10.1002/ece3.4795)

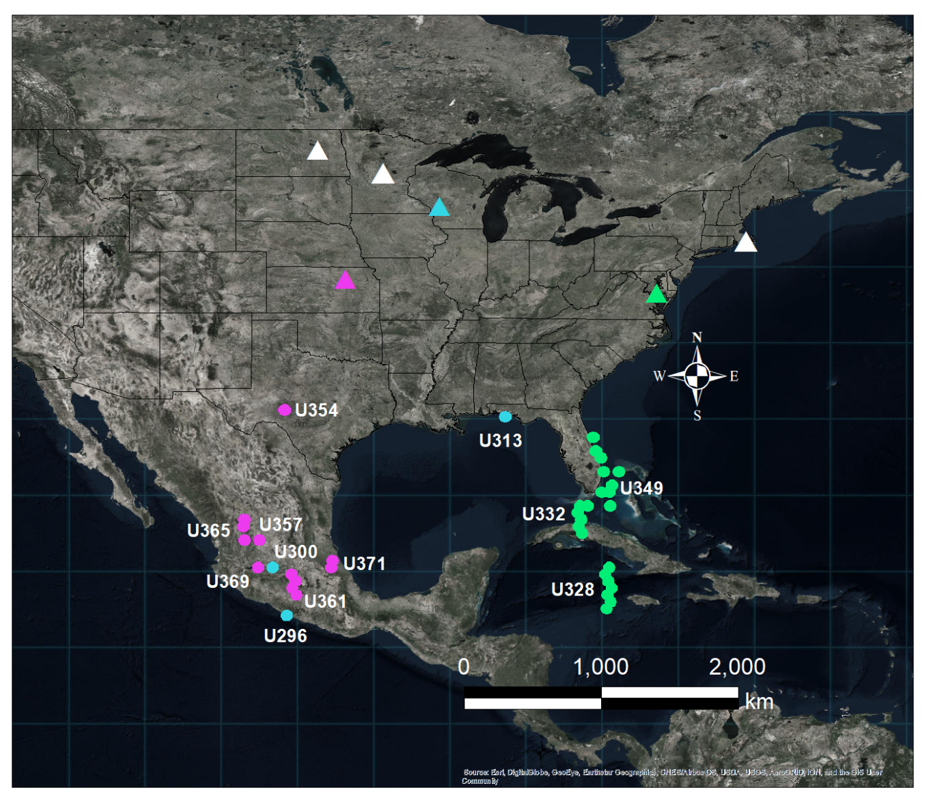

Supplement: Supplementary file 1 [file ECE3-9-680-s001.tif]

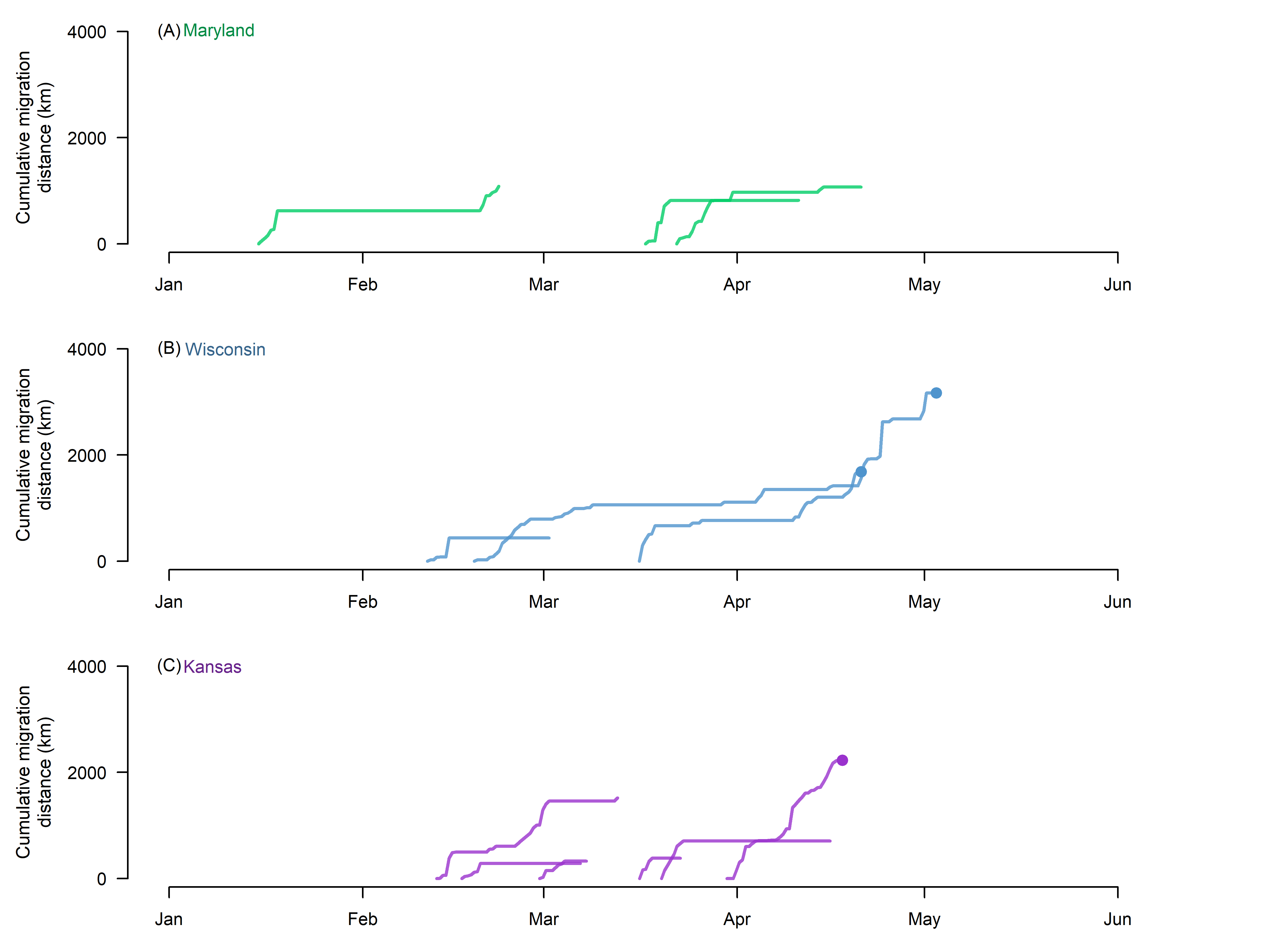

Supplement: Supplementary file 2 [file ECE3-9-680-s002.tiff]
